# Supplementary material for: Negative Vaccine Attitudes and Intentions to Vaccinate Against Covid-19 in Relation to Smoking Status: A Population Survey of UK Adults
Source: Nicotine Tob Res. 2021 Mar 5;23(9):1623–8. doi: 10.1093/ntr/ntab039 (PMC7989175; doi:10.1093/ntr/ntab039)
Supplement: ntab039_suppl_Supplementary_File_1 [file ntab039_suppl_supplementary_file_1.docx]

**Supplementary File 1: Imputed results**

Multiple imputation was used to generate 10 imputed datasets for each variable in participants with complete data on all vaccine attitudes and intent (*n* = 32,366). Imputation models included all variables used in the analysis. Pooled results are reported in the tables below.

| **Table 1.** Sample characteristics in relation to smoking status: imputed data | | | | |  |
| --- | --- | --- | --- | --- | --- |
|  | | **Never smokers**  **(*n*=20528)** | **Former smokers**  **(*n*=8067)** | **Current smokers**  **(*n*=3771)** | ***p*** |
| Mean (SE) age | | 46.5 (0.12) | 54.0 (0.17) | 48.3 (0.23) | <0.001 |
| % female | | 53.4 | 44.7 | 47.8 | <0.001 |
| % ethnic minority groups | | 17.1 | 8.1 | 14.4 | <0.001 |
| % income | |  |  |  | <0.001 |
|  | <£16,000 | 16.7 | 21.0 | 32.2 | - |
|  | £16,000-29,999 | 27.1 | 30.0 | 31.0 | - |
|  | £30,000-59,999 | 33.6 | 33.0 | 24.6 | - |
|  | £60,000-89,999 | 13.2 | 10.4 | 8.3 | - |
|  | ≥£90,000 | 9.5 | 5.6 | 3.9 | - |
| % key worker | | 20.1 | 19.8 | 27.8 | <0.001 |
| % ≥1 chronic physical health condition | | 33.6 | 47.3 | 41.7 | <0.001 |
| SD, standard deviation.  All data are weighted to match the UK population on gender, age, ethnicity, education, and country of living. | | | | | |

| **Table 2.** Associations between smoking status and negative attitudes towards vaccines: imputed data | | | | | | | | | | | | | | | | |
| --- | --- | --- | --- | --- | --- | --- | --- | --- | --- | --- | --- | --- | --- | --- | --- | --- |
|  | | **Mistrust of vaccine benefits** | | |  | **Worries about unforeseen future effects** | | |  | **Concerns about commercial profiteering** | | |  | **Preference for natural immunity** | | |
| **Descriptive data** | | **Mean^1^** | **SE** | **% high^2^** |  | **Mean^1^** | **SE** | **% high^2^** |  | **Mean^1^** | **SE** | **% high^2^** |  | **Mean^1^** | **SE** | **% high^2^** |
|  | Never smoker | 2.07 | 0.01 | 5.9 |  | 3.46 | 0.01 | 18.0 |  | 2.42 | 0.01 | 7.3 |  | 2.85 | 0.01 | 8.4 |
|  | Former smoker | 2.26 | 0.02 | 8.8 |  | 3.58 | 0.01 | 20.1 |  | 2.68 | 0.02 | 10.6 |  | 3.10 | 0.02 | 11.4 |
|  | Current smoker | 2.49 | 0.02 | 12.1 |  | 3.76 | 0.02 | 24.4 |  | 2.91 | 0.02 | 11.1 |  | 3.22 | 0.02 | 11.1 |
| **Linear regressions** | | ***B*** | **95% CI** | ***P*** |  | ***B*** | **95% CI** | ***p*** |  | ***B*** | **95% CI** | ***p*** |  | ***B*** | **95% CI** | ***p*** |
| Model 1^3^ | |  |  |  |  |  |  |  |  |  |  |  |  |  |  |  |
|  | Former smoker  *[ref never smoker]* | 0.19 | 0.16; 0.22 | <0.001 |  | 0.12 | 0.09; 0.15 | <0.001 |  | 0.27 | 0.23; 0.30 | <0.001 |  | 0.25 | 0.22; 0.29 | <0.001 |
|  | Current smoker  *[ref never smoker]* | 0.42 | 0.37; 0.46 | <0.001 |  | 0.30 | 0.26; 0.35 | <0.001 |  | 0.49 | 0.45; 0.54 | <0.001 |  | 0.37 | 0.32; 0.41 | <0.001 |
|  | Current smoker  *[ref former smoker]* | 0.23 | 0.18; 0.28 | <0.001 |  | 0.18 | 0.13; 0.23 | <0.001 |  | 0.23 | 0.17; 0.28 | <0.001 |  | 0.11 | 0.06; 0.17 | <0.001 |
| Model 2^4^ | |  |  |  |  |  |  |  |  |  |  |  |  |  |  |  |
|  | Former smoker  *[ref never smoker]* | 0.18 | 0.14; 0.21 | <0.001 |  | 0.05 | 0.02; 0.08 | 0.003 |  | 0.21 | 0.17; 0.24 | <0.001 |  | 0.11 | 0.07; 0.14 | <0.001 |
|  | Current smoker  *[ref never smoker]* | 0.31 | 0.27; 0.36 | <0.001 |  | 0.22 | 0.17; 0.26 | <0.001 |  | 0.35 | 0.30; 0.39 | <0.001 |  | 0.26 | 0.21; 0.31 | <0.001 |
|  | Current smoker  *[ref former smoker]* | 0.14 | 0.09; 0.19 | <0.001 |  | 0.17 | 0.12; 0.21 | <0.001 |  | 0.14 | 0.09; 0.19 | <0.001 |  | 0.15 | 0.10; 0.20 | <0.001 |
| Model 3^5^ | |  |  |  |  |  |  |  |  |  |  |  |  |  |  |  |
|  | Former smoker  *[ref never smoker]* | 0.18 | 0.15; 0.22 | <0.001 |  | 0.05 | 0.02; 0.08 | 0.002 |  | 0.21 | 0.17; 0.25 | <0.001 |  | 0.12 | 0.08; 0.15 | <0.001 |
|  | Current smoker  *[ref never smoker]* | 0.32 | 0.27; 0.36 | <0.001 |  | 0.22 | 0.17; 0.26 | <0.001 |  | 0.35 | 0.30; 0.40 | <0.001 |  | 0.27 | 0.22; 0.32 | <0.001 |
|  | Current smoker  *[ref former smoker]* | 0.14 | 0.09; 0.19 | <0.001 |  | 0.17 | 0.12; 0.21 | <0.001 |  | 0.14 | 0.09; 0.19 | <0.001 |  | 0.15 | 0.10; 0.20 | <0.001 |
| ^1^ Possible range 1-6.  ^2^ Score of 5 or 6.  ^3^ Unadjusted.  ^4^ Adjusted for age, gender, ethnicity, income, and key worker status.  ^5^ Adjusted for age, gender, ethnicity, income, key worker status, and chronic physical health conditions.  All data are weighted to match the UK population on gender, age, ethnicity, education, and country of living. | | | | | | | | | | | | | | | | |

| **Table 3.** Associations between smoking status and uncertainty and unwillingness to vaccinate against Covid-19: imputed data | | | | | | | | | |
| --- | --- | --- | --- | --- | --- | --- | --- | --- | --- |
|  | | **Undecided** | | |  | **Unwilling** | | | |
| **Descriptive data** | | **%** | **95% CI** | **-** |  | **%** | **95% CI** | **-** |  |
|  | Never smoker | 22.7 | 22.1; 23.3 | - |  | 12.1 | 11.6; 12.5 | - |  |
|  | Former smoker | 19.5 | 18.6; 20.3 | - |  | 15.1 | 14.3; 15.9 | - |  |
|  | Current smoker | 27.5 | 26.0; 28.9 | - |  | 21.8 | 20.5; 23.1 | - |  |
| **Multinomial logistic regressions** | | **RR** | **95% CI** | ***p*** |  | **RR** | **95% CI** | ***p*** |  |
| Model 1^1^ | |  |  |  |  |  |  |  |  |
|  | Former smoker  *[ref never smoker]* | 0.86 | 0.80; 0.91 | <0.001 |  | 1.25 | 1.16; 1.35 | <0.001 |  |
|  | Current smoker  *[ref never smoker]* | 1.55 | 1.43; 1.69 | <0.001 |  | 2.32 | 2.11; 2.54 | <0.001 |  |
|  | Current smoker  *[ref former smoker]* | 1.82 | 1.65; 2.00 | <0.001 |  | 1.85 | 1.67; 2.06 | <0.001 |  |
| Model 2^2^ | |  |  |  |  |  |  |  |  |
|  | Former smoker  *[ref never smoker]* | 0.89 | 0.84; 0.96 | 0.001 |  | 1.34 | 1.24; 1.45 | <0.001 |  |
|  | Current smoker  *[ref never smoker]* | 1.38 | 1.27; 1.51 | <0.001 |  | 1.99 | 1.80; 2.19 | <0.001 |  |
|  | Current smoker  *[ref former smoker]* | 1.55 | 1.40; 1.71 | <0.001 |  | 1.49 | 1.33; 1.66 | <0.001 |  |
| Model 3^3^ | |  |  |  |  |  |  |  |  |
|  | Former smoker  *[ref never smoker]* | 0.92 | 0.86; 0.98 | 0.013 |  | 1.38 | 1.27; 1.49 | <0.001 |  |
|  | Current smoker  *[ref never smoker]* | 1.43 | 1.31; 1.55 | <0.001 |  | 2.05 | 1.86; 2.27 | <0.001 |  |
|  | Current smoker  *[ref former smoker]* | 1.55 | 1.41; 1.71 | <0.001 |  | 1.49 | 1.34; 1.66 | <0.001 |  |
| ^1^ Unadjusted.  ^2^ Adjusted for age, gender, ethnicity, income, and key worker status.  ^3^ Adjusted for age, gender, ethnicity, income, key worker status, and chronic physical health conditions.  All data are weighted to match the UK population on gender, age, ethnicity, education, and country of living. | | | | | | | | | |
