# Supplementary material for: Negative Vaccine Attitudes and Intentions to Vaccinate Against Covid-19 in Relation to Smoking Status: A Population Survey of UK Adults
Source: Nicotine Tob Res. 2021 Mar 5;23(9):1623–8. doi: 10.1093/ntr/ntab039 (PMC7989175; doi:10.1093/ntr/ntab039)
Supplement: ntab039_suppl_Supplementary_File_3 [file ntab039_suppl_supplementary_file_3.docx]

**Supplementary File 3: Sample characteristics**

| **Table 1.** Sample characteristics in relation to smoking status | | | | |  |
| --- | --- | --- | --- | --- | --- |
|  | | **Never smokers**  **(*n*=17856)** | **Former smokers**  **(*n*=7285)** | **Current smokers**  **(*n*=3488)** | ***p*** |
| Mean (SD) age | | 47.0 (17.1) | 53.8 (15.0) | 46.2 (14.0) | <0.001 |
| % female | | 52.2 | 45.2 | 47.4 | <0.001 |
| % ethnic minority groups | | 14.0 | 7.7 | 14.9 | <0.001 |
| % income | |  |  |  | <0.001 |
|  | <£16,000 | 16.4 | 20.8 | 32.0 | - |
|  | £16,000-29,999 | 27.2 | 30.0 | 30.9 | - |
|  | £30,000-59,999 | 33.7 | 33.1 | 24.9 | - |
|  | £60,000-89,999 | 13.1 | 10.4 | 8.3 | - |
|  | ≥£90,000 | 9.6 | 5.7 | 3.9 | - |
| % key worker | | 21.2 | 20.4 | 28.7 | <0.001 |
| % ≥1 chronic physical health condition | | 33.7 | 46.4 | 41.4 | <0.001 |
| SD, standard deviation.  All data are weighted to match the UK population on gender, age, ethnicity, education, and country of living. | | | | | |
