# Supplementary material for: Negative Vaccine Attitudes and Intentions to Vaccinate Against Covid-19 in Relation to Smoking Status: A Population Survey of UK Adults
Source: Nicotine Tob Res. 2021 Mar 5;23(9):1623–8. doi: 10.1093/ntr/ntab039 (PMC7989175; doi:10.1093/ntr/ntab039)
Supplement: ntab039_suppl_Supplementary_File_4 [file ntab039_suppl_supplementary_file_4.docx]

**Supplementary File 4: Stratified results**

| **Table 1.** Associations between smoking status and uncertainty and unwillingness to vaccinate against Covid-19: stratified by age group | | | | | | | | | | | | |  |  |
| --- | --- | --- | --- | --- | --- | --- | --- | --- | --- | --- | --- | --- | --- | --- |
| **Age <60y** | |  |  |  | | | | | | |  | | |  |
|  | | **Intend to vaccinate** | | **Undecided** | | |  | **Unwilling** | | |  | | |  |
| **Descriptive data** | | **%** | **95% CI** | **%** | **95% CI** | **-** |  | **%** | **95% CI** | **-** | |  | | |
|  | Never smoker | 66.0 | 64.7; 67.2 | 24.1 | 23.0; 25.3 | - |  | 9.9 | 9.1; 10.7 | - | |  | | |
|  | Former smoker | 61.6 | 58.4; 64.7 | 19.3 | 16.8; 21.9 | - |  | 19.1 | 16.6; 21.7 | - | |  | | |
|  | Current smoker | 45.8 | 42.3; 49.3 | 28.3 | 25.2; 31.4 | - |  | 25.9 | 22.9; 29.0 | - | |  | | |
| **Multinomial logistic regressions^1^** | | **-** | **-** | **RR** | **95% CI** | ***p*** |  | **RR** | **95% CI** | ***p*** | |  | | |
|  | Former smoker  *[ref never smoker]* | - | - | 0.89 | 0.74; 1.07 | 0.200 |  | 2.11 | 1.73; 2.58 | <0.001 | |  | | |
|  | Current smoker  *[ref never smoker]* | - | - | 1.45 | 1.21; 1.75 | <0.001 |  | 3.28 | 2.68; 4.02 | <0.001 | |  | | |
|  | Current smoker  *[ref former smoker]* | - | - | 1.64 | 1.29; 2.09 | <0.001 |  | 1.56 | 1.21; 1.99 | <0.001 | |  | | |
| **Age ≥60y** | |  | |  | | |  |  | | |  | | |  |
|  | | **Intend to vaccinate** | | **Undecided** | | |  | **Unwilling** | | |  | | |  |
| **Descriptive data** | | **%** | **95% CI** | **%** | **95% CI** | **-** |  | **%** | **95% CI** | **-** | |  | | |
|  | Never smoker | 71.6 | 70.3; 72.8 | 19.0 | 17.9; 20.1 | - |  | 9.4 | 8.6; 10.2 | - | |  | | |
|  | Former smoker | 74.5 | 72.9; 76.0 | 15.6 | 14.3; 16.9 | - |  | 9.9 | 8.8; 11.0 | - | |  | | |
|  | Current smoker | 59.3 | 55.6; 63.1 | 27.3 | 23.8; 30.7 | - |  | 13.4 | 10.8; 16.0 | - | |  | | |
| **Multinomial logistic regressions^1^** | | **-** | **-** | **RR** | **95% CI** | ***p*** |  | **RR** | **95% CI** | ***p*** | |  | | |
|  | Former smoker  *[ref never smoker]* | - | - | 0.79 | 0.70; 0.89 | <0.001 |  | 1.00 | 0.85; 1.17 | 0.987 | |  | | |
|  | Current smoker  *[ref never smoker]* | - | - | 1.43 | 1.18; 1.75 | <0.001 |  | 1.28 | 0.99; 1.65 | 0.065 | |  | | |
|  | Current smoker  *[ref former smoker]* | - | - | 1.82 | 1.48; 2.24 | <0.001 |  | 1.28 | 0.98; 1.67 | 0.074 | |  | | |
| ^1^ Adjusted for age, gender, ethnicity, income, key worker status, and chronic physical health conditions.  All data are weighted to match the UK population on gender, age, ethnicity, education, and country of living. | | | | | | | | | | | | |  |  |

| **Table 2.** Associations between smoking status and uncertainty and unwillingness to vaccinate against Covid-19: stratified by key worker status | | | | | | | | | | | | |  |  |
| --- | --- | --- | --- | --- | --- | --- | --- | --- | --- | --- | --- | --- | --- | --- |
| **Not a key worker** | |  |  |  | | | | | | |  | | |  |
|  | | **Intend to vaccinate** | | **Undecided** | | |  | **Unwilling** | | |  | | |  |
| **Descriptive data** | | **%** | **95% CI** | **%** | **95% CI** | **-** |  | **%** | **95% CI** | **-** | |  | | |
|  | Never smoker | 67.3 | 66.5; 68.1 | 21.8 | 21.1; 22.4 | - |  | 11.0 | 10.4; 11.5 | - | |  | | |
|  | Former smoker | 67.6 | 66.4; 68.8 | 19.1 | 18.1; 20.1 | - |  | 13.3 | 12.4; 14.2 | - | |  | | |
|  | Current smoker | 54.5 | 52.5; 56.4 | 24.8 | 23.1; 26.5 | - |  | 20.7 | 19.1; 22.3 | - | |  | | |
| **Multinomial logistic regressions^1^** | | **-** | **-** | **RR** | **95% CI** | ***p*** |  | **RR** | **95% CI** | ***p*** | |  | | |
|  | Former smoker  *[ref never smoker]* | - | - | 0.95 | 0.88; 1.03 | 0.219 |  | 1.35 | 1.94; 2.46 | <0.001 | |  | | |
|  | Current smoker  *[ref never smoker]* | - | - | 1.32 | 1.19; 1.47 | <0.001 |  | 2.19 | 1.94; 2.46 | <0.001 | |  | | |
|  | Current smoker  *[ref former smoker]* | - | - | 1.39 | 1.23; 1.57 | <0.001 |  | 1.63 | 1.43; 1.85 | <0.001 | |  | | |
| **Key worker** | |  | |  | | |  |  | | |  | | |  |
|  | | **Intend to vaccinate** | | **Undecided** | | |  | **Unwilling** | | |  | | |  |
| **Descriptive data** | | **%** | **95% CI** | **%** | **95% CI** | **-** |  | **%** | **95% CI** | **-** | |  | | |
|  | Never smoker | 60.0 | 58.4; 61.5 | 26.1 | 24.7; 27.5 | - |  | 14.0 | 12.9; 15.1 | - | |  | | |
|  | Former smoker | 59.6 | 57.1; 62.1 | 20.2 | 18.2; 22.3 | - |  | 20.2 | 18.1; 22.2 | - | |  | | |
|  | Current smoker | 42.1 | 39.0; 45.2 | 34.4 | 31.4; 37.3 | - |  | 23.6 | 20.9; 26.2 | - | |  | | |
| **Multinomial logistic regressions^1^** | | **-** | **-** | **RR** | **95% CI** | ***p*** |  | **RR** | **95% CI** | ***p*** | |  | | |
|  | Former smoker  *[ref never smoker]* | - | - | 0.80 | 0.68; 0.93 | 0.004 |  | 1.50 | 1.27; 1.77 | <0.001 | |  | | |
|  | Current smoker  *[ref never smoker]* | - | - | 1.71 | 1.45; 2.02 | <0.001 |  | 1.92 | 1.58; 2.33 | <0.001 | |  | | |
|  | Current smoker  *[ref former smoker]* | - | - | 2.14 | 1.76; 2.62 | <0.001 |  | 1.28 | 1.03; 1.59 | 0.026 | |  | | |
| ^1^ Adjusted for age, gender, ethnicity, income, and chronic physical health conditions.  All data are weighted to match the UK population on gender, age, ethnicity, education, and country of living. | | | | | | | | | | | | |  |  |

| **Table 3.** Associations between smoking status and uncertainty and unwillingness to vaccinate against Covid-19: stratified by chronic physical health conditions | | | | | | | | | | | | |  |  |
| --- | --- | --- | --- | --- | --- | --- | --- | --- | --- | --- | --- | --- | --- | --- |
| **No chronic physical health conditions** | |  |  |  | | | | | | |  | | |  |
|  | | **Intend to vaccinate** | | **Undecided** | | |  | **Unwilling** | | |  | | |  |
| **Descriptive data** | | **%** | **95% CI** | **%** | **95% CI** | **-** |  | **%** | **95% CI** | **-** | |  | | |
|  | Never smoker | 62.8 | 62.0; 63.7 | 24.6 | 23.8; 25.4 | - |  | 12.6 | 12.0; 13.2 | - | |  | | |
|  | Former smoker | 61.5 | 59.9; 63.0 | 21.0 | 19.8; 22.3 | - |  | 17.5 | 16.3; 18.7 | - | |  | | |
|  | Current smoker | 48.1 | 45.9; 50.2 | 29.1 | 27.2; 31.1 | - |  | 22.8 | 21.0; 24.6 | - | |  | | |
| **Multinomial logistic regressions^1^** | | **-** | **-** | **RR** | **95% CI** | ***p*** |  | **RR** | **95% CI** | ***p*** | |  | | |
|  | Former smoker  *[ref never smoker]* | - | - | 0.93 | 0.85; 1.02 | 0.124 |  | 1.51 | 1.36; 1.67 | <0.001 | |  | | |
|  | Current smoker  *[ref never smoker]* | - | - | 1.39 | 1.24; 1.55 | <0.001 |  | 2.07 | 1.82; 2.35 | <0.001 | |  | | |
|  | Current smoker  *[ref former smoker]* | - | - | 1.49 | 1.30; 1.70 | <0.001 |  | 1.37 | 1.19; 1.59 | <0.001 | |  | | |
| **≥1 chronic physical health condition** | |  | |  | | |  |  | | |  | | |  |
|  | | **Intend to vaccinate** | | **Undecided** | | |  | **Unwilling** | | |  | | |  |
| **Descriptive data** | | **%** | **95% CI** | **%** | **95% CI** | **-** |  | **%** | **95% CI** | **-** | |  | | |
|  | Never smoker | 71.5 | 70.3; 72.6 | 18.9 | 17.9; 19.9 | - |  | 9.7 | 8.9; 10.4 | - | |  | | |
|  | Former smoker | 71.2 | 69.7; 72.8 | 17.3 | 16.0; 18.6 | - |  | 11.5 | 10.4; 12.5 | - | |  | | |
|  | Current smoker | 54.9 | 52.4; 57.5 | 25.4 | 23.1; 27.6 | - |  | 19.7 | 17.6; 21.7 | - | |  | | |
| **Multinomial logistic regressions^1^** | | **-** | **-** | **RR** | **95% CI** | ***p*** |  | **RR** | **95% CI** | ***p*** | |  | | |
|  | Former smoker  *[ref never smoker]* | - | - | 0.91 | 0.81; 1.01 | 0.084 |  | 1.21 | 1.05; 1.39 | 0.010 | |  | | |
|  | Current smoker  *[ref never smoker]* | - | - | 1.52 | 1.32; 1.76 | <0.001 |  | 2.13 | 1.80; 2.51 | <0.001 | |  | | |
|  | Current smoker  *[ref former smoker]* | - | - | 1.68 | 1.44; 1.97 | <0.001 |  | 1.77 | 1.48; 2.11 | <0.001 | |  | | |
| ^1^ Adjusted for age, gender, ethnicity, income, and key worker status.  All data are weighted to match the UK population on gender, age, ethnicity, education, and country of living. | | | | | | | | | | | | |  |  |
